# Presentation and Treatment of a Combined Median Nerve Schwannoma and a C7 Discogenic Radiculopathy

**DOI:** 10.7759/cureus.3009

**Published:** 2018-07-20

**Authors:** Mahesh B Shenai, Geetha Menezes, Drew Falconer, James Leiphart

**Affiliations:** 1 Neurosurgery, Inova Fairfax, Vienna, USA; 2 Pathology, Inova Fairfax Hospital, Falls Church, USA; 3 Inova Parkinson's and Movement Disorders Program, Inova Health System, Falls Church, USA; 4 Inova Neurosurgery Department, Inova Neurscience Institute, Falls Church, USA

**Keywords:** schwannoma, cervical radiculopathy, double-crush, median nerve

## Abstract

Cervical radiculopathy and peripheral nerve pathology often compete in the differential diagnosis of extremity pain, weakness, and numbness, and frequently, coexist. In this report, we describe a 73-year-old male with a previously asymptomatic left anteromedial proximal upper arm mass, who presented with progressive radicular arm pain, proximal and distal upper extremity weakness, and hand numbness. Clinical investigation revealed a prominent C6-7 disc herniation and a median nerve sheath tumor, with electromyography (EMG)/nerve conduction velocity (NCV) studies suggestive of acute radiculopathy. He was treated in a staged surgical fashion, with the nerve sheath tumor resection first, followed by a standard C6-7 anterior cervical discectomy and fusion (ACDF) two weeks later. The patient made a full recovery. We provide a literature review and discussion of the “double crush” hypothesis.

## Introduction

Cervical radiculopathy is a very common presentation in many neurosurgical and orthopedic clinics, presenting with a combination of radiating or shooting pain, dermatomal numbness, paresthesias, and myotomal weakness. Diagnosis of cervical radiculopathies are straightforward, especially when objective evidence exists from radiographic or electrophysiological studies. Peripheral nerve sheath tumors, on the other hand, are relatively rare and can be seen in association with neurofibromatosis. While often asymptomatic, progressive nerve sheath tumors can also present with paresthesias, pain, numbness, and weakness. The diagnosis is generally made with physical examination, combined with magnetic resonance imaging (MRI) or ultrasound, in combination with electrophysiological studies [1-2], although biopsy/excision is the only way to differentiate between types of nerve sheath tumors, such as schwannomas, neurofibromas, and malignant peripheral nerve sheath tumors (MPNST) [3]. Rarely, symptomatic cervical radiculopathies can be seen in conjunction with nerve sheath tumors, yielding an overlapping clinical presentation and concurrent management. The case below describes a patient with a previously asymptomatic median nerve schwannoma, who developed independent radicular symptoms that then instigated a concurrent acute median neuropathy.

## Case presentation

**Figure 1:**
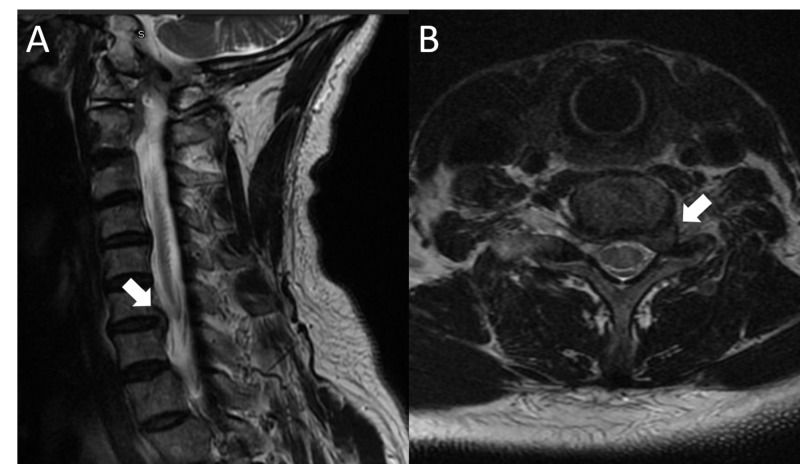
Preoperative cervical magnetic resonance imaging (MRI) (A) Sagittal T2 cervical MRI scan demonstrating a left C6-7 disc herniation. (B) Axial T2 cervical MRI at the C6-7 disc space, depicting a laterally protruding left disc herniation obstructing the neural foramen.

**Figure 2:**
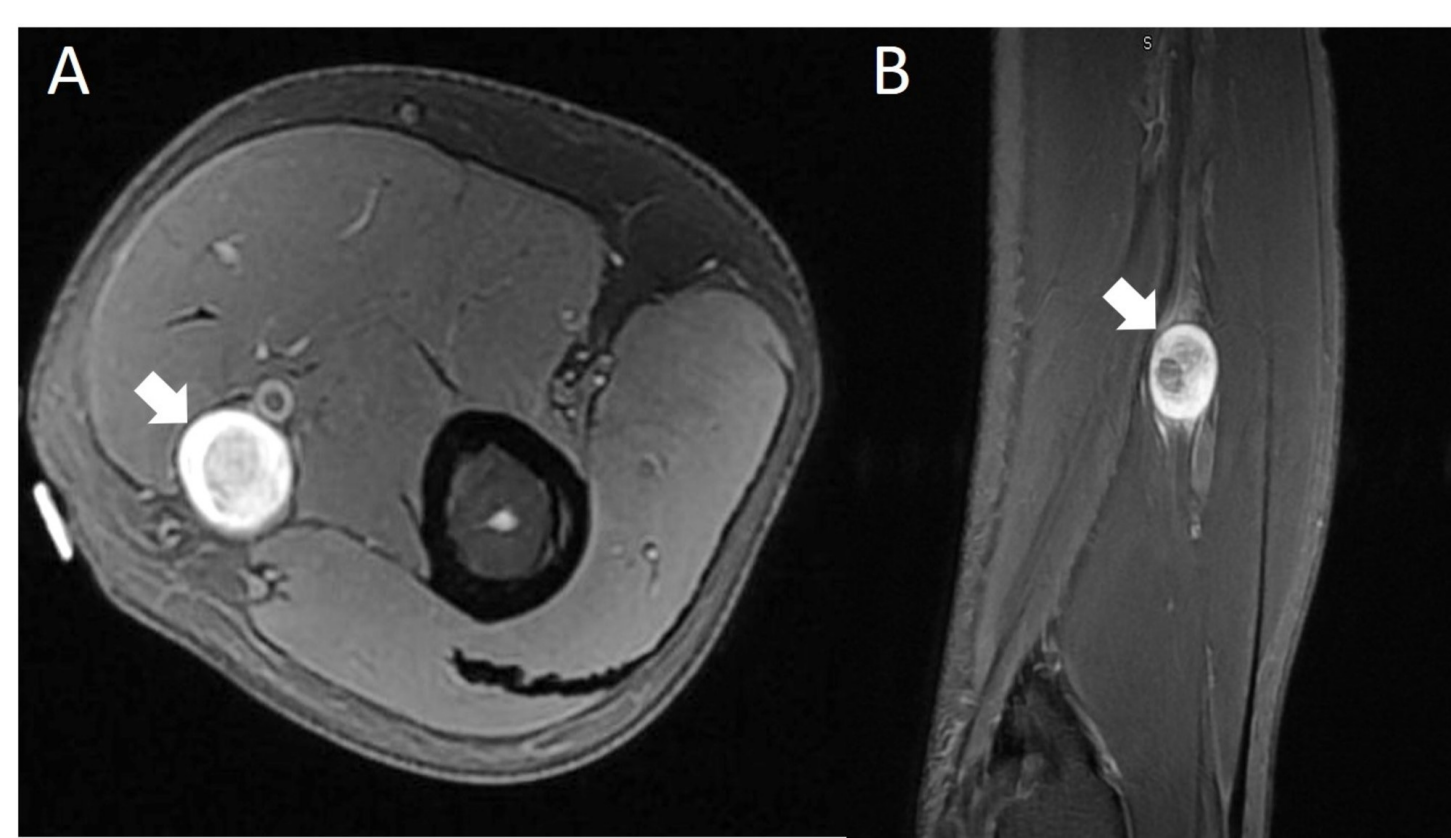
Magnetic resonance imaging (MRI) of the left upper extremity (A) Axial T1 MRI with contrast demonstrating spherical mass in the proximal anteromedial upper arm. (B) Sagittal T1 MRI with contrast demonstrating continuity with the median nerve.

The patient is a 73-year-old male, with only a past medical history of bilateral cataracts and hyperlipidemia. He had complained of one year of left-sided radiating neck pain into the shoulder, arm and the second digit, with numbness in the second digit. He was noted to have a palpable left anteromedial upper arm mass one year ago, which was subjected to needle biopsy and determined to be a “benign schwannoma”. Initially, the mass was painless, without a Tinel’s sign. Upon initial presentation to our clinic, his radicular pain was worsening. He also developed pain in the upper arm around the palpable mass, with shooting pain into the hand with percussion. There was a positive Tinel’s sign, with the percussion of the mass leading to pain and paresthesias in the left hand. On exam, the patient had 4/5 weakness in the left triceps, 4+/5 weakness in pronation, and a mild “benediction” sign, with incomplete voluntary flexion of the second and third digits. Sensory examination revealed reduced sensation to pinprick in the left palmar index finger and thumb. All deep tendon reflexes were diminished, but symmetric. Magnetic resonance imaging (MRI) of the cervical spine revealed a prominent left C6-7 foraminal disc protrusion, causing C7 nerve root compression as shown in Figure 1. An MRI of the left humerus revealed a circumscribed ellipsoid mass along the anteromedial distal upper arm, contiguous with the median nerve as demonstrated in Figure 2. Electromyography (EMG) and nerve conduction velocity (NCV) study of the upper extremities revealed acute and chronic denervation changes in the left flexor carpi radialis (FCR) with reduced recruitment in the triceps and the cervical paraspinal muscles.

The patient was counseled and offered a staged surgical approach, with resection of the upper arm mass first, followed by a C6-7 anterior cervical discectomy and fusion (ACDF). The resection of the upper arm mass was performed under general anesthesia; through a linear anteromedial distal upper arm incision, the tumor capsule was identified and contiguous with the median nerve. Intraoperative monitoring and stimulation were performed and a capsular incision was made in a region on no activity. The tumor was resected with a fascicle entering and exiting the tumor, without nerve action potential transmission. After resection, the epineurium was closed with 6-0 prolene, and the skin was closed in a layered fashion. Post-operatively, the patient’s motor examination was unchanged, but his pain had significantly improved. He returned to the operating room two weeks later for the C6-7 ACDF, which included a full discectomy and division of the posterior longitudinal ligament, with direct decompression of the left C7 nerve root, performed with use of sensory and motor evoked potentials, and EMGs via intraoperative monitoring.

**Figure 3:**
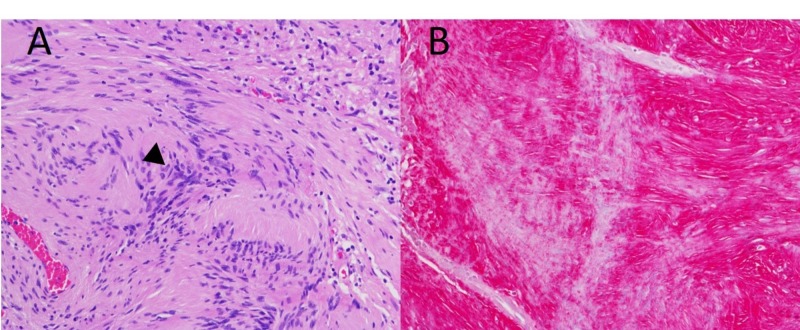
Pathological examination of median nerve mass (A) Hematoxylin and eosin (H&E) stain at 200x magnification, demonstrating Verocay bodies (arrow); (B) S100 red immunostaining at 200x.

Pathological examination of the nerve sheath mass revealed a circumscribed spindle cell lesion demonstrating hypercellular areas alternating with hypocellular areas (Antoni A and B regions), in addition to Verocay bodies shown in Figure 3A. S100 red immunostaining confirmed the diagnosis of a peripheral schwannoma, as depicted in Figure 3B.

By four weeks post-operatively, his triceps function returned to full strength, as did his strength in the finger flexion of his index finger. Numbness persisted in the left thumb and index finger, although it improved in its intensity. The neck and radiating arm pain resolved completely. The only complication was post-operative hoarseness of the voice, which also improved by three months post-operatively.

## Discussion

The above patient presents an intriguing scenario of a coexisting radicular and peripheral nerve pathologies, resulting in a mixed presentation and examination. To our knowledge, this is the first published report of a coexisting proximal median nerve sheath tumor and a discogenic radiculopathy. The patient presented for surgical consideration primarily due to the progressive radicular pain and triceps weakness, exclusively explained by the C7 discogenic nerve root compression. However, weakness and electrophysiologic abnormality of the FCR could originate either from the C7 compression or intrinsic median nerve pathology, as shown in Table 1 [4]. The numbness of the palmar thumb and index finger also represent an overlapping region of the C7 dermatome and the median nerve region of innervation. The presence of the Tinel’s sign clearly implicates the irritability of the nerve sheath tumor and the nerve’s contribution to dysfunction. Surgical attention to both sources, the radicular decompression and tumor resection, were both required to optimally treat this patient. Failure to diagnose or treat either pathology would have resulted in residual symptoms. If on the other hand there was no triceps weakness and only FCR dysfunction, then it would be reasonable to only address the median nerve pathology, and defer on the ACDF.

**Table 1:** C7 and median nerve innervations Denotes muscles of joint C7/median nerve innervation. ^*^Data taken from [4]​​​​​​​.

	C7 Nerve Root	Median Nerve
Myotomal Distribution	Triceps Brachii	
	Palmaris Longus^*^	Palmaris Longus^*^
	Flexor Carpi Radialis^*^	Flexor Carpi Radialis^*^
	Pronator Teres^*^	Pronator Teres^*^
	Extensor Carpi Radialis Longus/Brevis	
	Extensor Carpi Ulnaris	Flexor digitorum superficialis
	Extensor Digitorum	Flexor digitorum profundus (1/2)
	Extensor Indicis	Flexor pollicis longus
	Extensor Pollicis Longus/Brevis	Pronator Quadratus
	Extensor Digiti Minimi	
	Supinator	
	Abductor Pollicis Longus	
Impairment in Case patient	Triceps Brachii	Flexor Carpi Radialis
	Flexor Carpi Radialis	Pronator Teres
	Pronator teres	

The presence of two or more pathologic insults along a common neuronal pathway has been described as a “double-crush” phenomena, whereby one lesion is hypothesized to predispose or potentiate the impact of other lesions. The underlying theory involves that serial insults to the neuronal pathway are additive, and restrict axonoplasmic flow. The insults can stem from compression, but can also be defined to include other pathologies, such as diabetic or autoimmune neuropathies. A number of descriptions and reviews exist in the literature [5-9]. The true existence of “double-crush” however, remains controversial [7]. We are unaware of any review or case report that describes the phenomena with respect to a cervical radiculopathy and a nerve sheath tumor, however, Raps et al. describe two rare cases of proximal median nerve neuropathy in conjunction with cervical radiculopathy [10]. In our patient, the initial lack of symptoms in the presence of a palpable mass, and later contributing to a mixed presentation, support the notion of a “double-crush” phenomena.

In the current patient, we elected to treat the peripheral nerve sheath tumor first, followed by the cervical discectomy at a later time. While there is no evidence for a recommendation on prioritizing or interval timing of these surgeries, we started with the peripheral tumor resection first, due to its greater potential for progression and the belief that resection had a lower risk of non-neurological complications, compared to those possible with an ACDF. Alternately, we believe it is reasonable to consider the ACDF first, as the nerve root compression can be better implicated in a greater number of signs and symptoms, and may have a better chance of resolving all symptoms. Nevertheless, with recently demonstrated growth over a short period of time, the presence of a positive Tinel's sign, and local pain, excision of the tumor was recommended in preference to continued observation. In either strategy, careful counseling of the patient and their family is critical, in managing expectations and satisfaction.

## Conclusions

Coexisting pathologies of the spinal nerve root and peripheral nerves produce challenging clinical scenarios, confounding the explanatory diagnosis, and the prioritization of treatment. In the patient presented above, a discogenic C7 radiculopathy and a proximal median nerve sheath tumor produced a mixed constellation of signs and symptoms implicating both pathologies, as hypothesized by the “double crush” phenomena. In similar scenarios, we encourage comprehensive work-up and discussion with the patient, ultimately encouraging addressing both pathologies in a staged surgical fashion, although the prioritization would depend on the nuances of the presentation.
